# Electrospun Alginate Fibers: Mixing of Two Different Poly(ethylene oxide) Grades to Improve Fiber Functional Properties

**DOI:** 10.3390/nano8120971

**Published:** 2018-11-25

**Authors:** Barbara Vigani, Silvia Rossi, Giulia Milanesi, Maria Cristina Bonferoni, Giuseppina Sandri, Giovanna Bruni, Franca Ferrari

**Affiliations:** 1 Department of Drug Sciences, University of Pavia, 27100 Pavia, Italy; barbara.vigani@unipv.it (B.V.); giulia.milanesi02@universitadipavia.it (G.M.); cbonferoni@unipv.it (M.C.B.); giuseppina.sandri@unipv.it (G.S.); franca.ferrari@unipv.it (F.F.); 2 Department of Chemistry, University of Pavia, 27100 Pavia, Italy; giovanna.bruni@unipv.it

**Keywords:** electrospinning, ALG-containing fibers, poly(ethylene oxide), molecular weight, critical entanglement concentration, mechanical properties, intermolecular hydrogen bonds

## Abstract

The aim of the present work was to investigate how the molecular weight (MW) of poly(ethylene oxide) (PEO), a synthetic polymer able to improve alginate (ALG) electrospinnability, could affect ALG-based fiber morphology and mechanical properties. Two PEO grades, having different MWs (high, h-PEO, and low, l-PEO) were blended with ALG: the concentrations of both PEOs in each mixture were defined so that each h-PEO/l-PEO combination would show the same viscosity at high shear rate. Seven ALG/h-PEO/l-PEO mixtures were prepared and characterized in terms of viscoelasticity and conductivity and, for each mixture, a complex parameter rH/rL was calculated to better identify which of the two PEO grades prevails over the other in terms of exceeding the critical entanglement concentration. Thereafter, each mixture was electrospun by varying the process parameters; the fiber morphology and mechanical properties were evaluated. Finally, viscoelastic measurements were performed to verify the formation of intermolecular hydrogen bonds between the two PEO grades and ALG. rH/rL has been proved to be the parameter that better explains the effect of the electrospinning conditions on fiber dimension. The addition of a small amount of h-PEO to l-PEO was responsible for a significant increase in fiber mechanical resistance, without affecting the nano-scale fiber size. Moreover, the mixing of h-PEO and l-PEO improved the interaction with ALG, resulting in an increase in chain entanglement degree that is functional in the electrospinning process.

## 1. Introduction

In the last two decades, electrospinning has been recognized as a simple, low-cost, and versatile method to obtain submicron fibers for a variety of biomedical applications, such as tissue engineering [1,2,3], drug delivery [4], and wound healing [5,6]. The recent success of such electrospun membranes relies on their peculiar architecture: they mimic the three-dimensional structure of the native extracellular matrix (ECM) of most human tissues and can be tailored according to the therapeutic need [7,8,9].

The electrospinning technique exploits electrostatic forces to produce fibers with a diameter from nano- to micro-scale starting from polymer solutions or blends. The apparatus setup consists of a syringe with a needle tip (spinneret), a high-voltage power supply, and a grounded collector, which is usually a plate or a cylindrical rotating drum [10]. During the process, the polymer solution or blend, loaded in the syringe, is subjected to an electric field applied between the spinneret and the collector. Such an electric field induces the formation of electric charges on the liquid surface, causing a deformation of the polymeric drop at the end of the needle tip from a spherical to a conical shape (Taylor’s cone). When the repulsive electrical forces overcome the surface tension of the polymer solution, a charged jet departs from the Taylor’s cone and deforms itself according to the electric field direction; simultaneously, the solvent evaporates, leaving dried fibers on the collector [11,12].

It is generally recognized that many variables can affect the electrospinning process: polymer solution properties (viscosity, surface tension, and conductivity), process parameters (applied voltage, spinneret–collector distance, and flow rate), and environmental conditions (temperature and relative humidity) play a pivotal role in the definition of fiber morphology and orientation and, thus, an in-depth study is required to establish the best electrospinning conditions for a specific polymeric solution or blend [13]. Additionally, polymer solution properties, in particular, viscosity and conductivity, are strongly affected by the solvent employed. A typical example was provided by Lee et al. [14], who studied the effect of solvent nature on poly(ε-caprolactone) (PCL) fiber morphology. They demonstrated that the addition of *N*,*N*-dimethylformamide to methylene chloride enhanced PCL electrospinnability and markedly reduced fiber diameter. This was attributable to the high dielectric constant and conductivity of the solution.

The versatility of such a method lies in the possibility of spinning more than 200 polymers, both synthetic and natural ones [11]. Among the latter, polysaccharides have attracted great interest thanks to their natural abundance, biocompatibility, biodegradability, low immunogenicity, and better clinical functionality, and some of them (alginate, chitosan, dextran, cellulose, hyaluronic acid, and starch) have recently become widely popular in the electrospinning field [15]. Despite this, the electrospinning of polysaccharides is still challenging, particularly due to their inability to form in water a sufficient number of chain entanglements to promote fiber formation [16,17]. The strategy most widely used to overcome this problem is blending polysaccharides with a second synthetic polymer, generally nontoxic and nonionic (i.e., poly(ethylene oxide) (PEO) or poly(vinyl alcohol) (PVA)), in order to improve their electrospinnability, thus preserving their biocompatibility [18,19].

Up to today, many research groups have exploited such a strategy to produce homogenous fibers based on alginate (ALG), which is an anionic polysaccharide, generally extracted from marine brown algae and widely used in skin, cartilage, bone, and cardiac tissue regeneration. ALG is a linear copolymer composed by blocks of α-l-guluronic acid (G) and β-d-mannuronic acid residues linked by a β-1,4-glycosidic bond, whose structure resembles that of glycosaminoglycans, which are the main components of the ECM [20].

In the last decade, several hypotheses have been formulated to explain the failures in electrospinning pure ALG.

An ALG aqueous solution starts to gel at concentrations equal to or greater than 2% w/v; concentrations lower than 2% w/v are too low for the production of continuous fibers, while an increase in ALG concentration generates solutions with a viscosity so high as to clog the spinneret. Another factor hindering the electrospinning process is that ALG is a polyelectrolyte: ALG aqueous solutions are therefore characterized by a high conductivity, which prevents the ejection of a stabilized jet through the needle. Finally, the ALG rigid and extended worm-like structure does not allow the formation of effective chain entanglement in aqueous solution, impeding polymer spinnability [21]. Focusing on the abovementioned issues, different research groups proposed the addition, as process adjuvant, of a copolymer or a co-solvent to ALG aqueous solution [22,23,24,25,26]. In particular, PEO has been selected because it can interact with ALG through hydrogen bonds, reducing the solution viscosity and modulating the repulsive forces among the polyanions while improving the flexibility of ALG chains [17,18,24,25,26].

To the best of our knowledge, up to now, no systematic research focused on the influence of the molecular weight (MW) of PEO on the morphology and mechanical properties of ALG-based fibers has been performed. In particular, it seems interesting to evaluate whether the use of mixtures of different PEO grades (having different MWs) could modulate fiber morphology and mechanical properties.

Given these premises, the aim of the present work was to investigate how the MW of PEO, when blended with ALG, could affect electrospun fiber morphology and mechanical properties; in particular, in order to design ALG-containing fibers with a nano-scale diameter, PEO with high MW (h-PEO), successfully used in a previous work to obtain micro-scale fibers [26], was substituted by PEO with low MW (l-PEO). In the attempt to modulate fiber morphology and to improve their mechanical properties, mixtures of h-PEO/l-PEO were used: the concentrations of both PEOs in each mixture were defined so that each h-PEO/l-PEO combination would show the same viscosity at high shear rate.

Polymer mixtures were characterized in terms of viscoelasticity and conductivity in order to evaluate the role of each property in defining the fiber morphology. Moreover, a complex parameter which takes into account for each PEO grade the ratio between the polymer concentration and the critical entanglement concentration (CEC) was considered to assess the contribution of polymer MW to the mixture properties that were functional in fiber formation.

Thereafter, each mixture was electrospun by varying the spinneret–collector distance and the applied voltage; scanning electron microscopy was exploited to investigate how the process conditions could affect fiber morphology (uniformity and dimensions). Mechanical properties of the fibers were also evaluated.

Finally, viscoelastic measurements were performed to assess the influence of PEO MW on the polymer capability to interact with ALG, increasing the chain entanglement and, in turn, improving the electrospinning process.

## 2. Materials and Methods

### 2.1. Materials

Alginic acid sodium salt from brown algae (ALG, medium viscosity, Brookfield viscosity ≥ 2000 cps (2% in water at 25 °C), G/M ratio of 70:30, Sigma Aldrich, St. Louis, MO, USA), poly(ethylene oxide) of high molecular weight (h-PEO, MW = 4000 kDa, Colorcon, Dartford, United Kingdom), poly(ethylene oxide) of low molecular weight (l-PEO, MW = 600 kDa, Sigma Aldrich, St. Louis, MO, USA), Kolliphor P407 (P407, BASF SE, Ludwigshafen, Germany), and Triton X-100 (TX100, Fluka BioChemika, Buchs, Switzerland) were used for the preparation of ALG/PEO solutions. 

### 2.2. Preparation of the Polymer Solutions for Electrospinning

#### 2.2.1. ALG/h-PEO Solution

An aqueous solution of ALG/h-PEO (H) was prepared according to [26]. Briefly, ALG and h-PEO (MW = 4000 kDa) were dissolved in deionized water to achieve concentrations equal to 1% and 1.5% *w*/*w*, respectively. Surfactants, in particular, P407 and TX100, were then added to the ALG/h-PEO blend at concentrations of 1.5% and 0.5% *w*/*w*, respectively. The solution was maintained under stirring overnight at room temperature before electrospinning.

#### 2.2.2. ALG/l-PEO Solution

A rheological study was performed to identify the concentration of l-PEO (MW = 600 kDa) that was iso-viscous with 1.5% *w*/*w* h-PEO in deionized water. Briefly, different l-PEO solutions with increasing polymer concentrations ranging from 1.5 to 5% *w*/*w* were prepared in deionized water, without surfactants. Rheological analyses were performed by means of a rotational rheometer (MCR102, Anton Paar, Turin, Italy), using a C50-1 cone (∅ = 50 mm and θ = 1°) as the measuring system. Viscosity measurements were performed in a shear rate range from 10 to 1000 s^−1^ at 33 °C (temperature to which the polymer solutions were subjected during the electrospinning process). Viscosity values, obtained at 1000 s^−1^ for each l-PEO solution, were plotted versus the corresponding polymer concentration (% *w*/*w*) on a bi-log scale. A fitted linear regression model was used. Knowing the viscosity at 1000 s^−1^ of a 1.5% *w*/*w* h-PEO aqueous solution (prepared without surfactants), it was possible to extrapolate the iso-viscous l-PEO concentration, which was equal to 2.2% *w*/*w*. This concentration was considered for the preparation of an aqueous solution of ALG/l-PEO (L). ALG and l-PEO were dissolved in deionized water to achieve concentrations equal to 1% and 2.2% *w*/*w*, respectively. Surfactants, in particular, P407 and TX100, were then added to the ALG/h-PEO blend at the concentrations of 1.5% and 0.5% *w*/*w*, respectively. The solution was maintained under stirring overnight at room temperature before being subjected to electrospinning.

#### 2.2.3. ALG/h-PEO/l-PEO Mixtures

Seven mixtures (M1–M7), containing 1% *w*/*w* ALG, h-PEO, and l-PEO were prepared in deionized water with the addition of P407 at 2% *w*/*w*; the concentrations of both PEO grades in each M were defined so that each h-PEO/l-PEO combination would have the same viscosity as a 1.5% *w*/*w* h-PEO aqueous solution (Table 1).

### 2.3. Characterization of the Electrospinning Solutions

All formulations (H, L, and Ms) were characterized in terms of viscoelastic properties and conductivity.

#### 2.3.1. Rheological Analysis

Rheological analyses were performed by means of a rotational rheometer (MCR102, Anton Paar, Turin, Italy), using a C50-1 cone (∅ = 50 mm and θ = 1°) as measuring system.

Sample viscoelasticity was assessed by dynamic oscillatory measurements such as stress sweep test and oscillation test. In the stress sweep test, increasing stresses were applied at a constant frequency (1 Hz), and the elastic response of the sample, expressed as storage modulus G′, was measured. Such a test allows us to identify the “linear viscoelastic region”. In the oscillation test, a shear stress, chosen in the linear viscoelastic region previously determined, was applied at increasing frequencies (0.1 to 10 Hz), and G′ and G″ profiles were recorded. Measurements were performed at 33 °C in triplicate.

#### 2.3.2. Conductivity Measurements

Conductivity measurements were carried out by means of a Mettler Toledo™ FiveGo™ F3 conductivity meter apparatus (Fisher Scientific, Milan, Italy). Three replicates were considered for each solution.

#### 2.3.3. Calculation of rH and rL Parameters

Two parameters, rH and rL, were calculated for each M according to the Equations (1) and (2), respectively:

rH = h-PEO concentration in M/h-PEO CEC(1)

rL = l-PEO concentration in M/l-PEO CEC(2)

For each M, the ratio rH/rL was also calculated.

### 2.4. Fiber Preparation and Morphological Characterization 

ALG/h-PEO and ALG/l-PEO fibers, named H and L, respectively, were prepared by using an electrospinning apparatus (STIKIT-40 Linari Engineering, Grosseto, Italy) equipped with a high-voltage power supply, a syringe pump, and a collector plate, covered by an aluminium foil. Different process parameters such as spinneret–collector distance (15–20 cm) and applied voltage (20–25 kV) were investigated in order to identify the best electrospinning conditions. The flow rate was equal to 0.8 mL/h.

Each mixture (M1–M7) was electrospun under four different process conditions (spinneret–collector distance, applied voltage), maintaining the flow rate equal to 0.8 mL/h: 15 cm, 15 kV; 20 cm, 20 kV; 20 cm, 25 kV; and 25 cm, 25 kV.

Polymer solutions were pumped through a needle (length 15 mm, gauge = 21), and the electrospinning process was performed at atmospheric pressure, maintaining temperature and relative humidity in the ranges 27–33 °C and 10–20%, respectively.

Morphological evaluation was performed by means of a scanning electron microscope (EVO MA10, Carl Zeiss, Oberkochen, Germany). Fiber size was measured using the imaging analysis program ImageJ 2.0. Thirty fibers were considered for each sample.

### 2.5. Assessment of Fiber Mechanical Properties

Fiber mechanical properties were assessed by means of a TA.XT plus Texture Analyzer (Stable Micro Systems, Godalming, United Kingdom), equipped with 5 kg load cells. Before testing, fiber thickness was measured by means of a Sicutool 3955G-50 (Milan, Italy) apparatus.

Each fiber was cut (1 cm × 3 cm) and then clamped on an A/TG tensile grips probe; an initial distance of 1 cm between the grips was set. The upper grip was raised at a constant speed of 5 mm/s up to a distance of 5 mm, corresponding to 50% elongation.

Maximum deformation force (Fmax; corresponding to force at break) and forces measured at different deformations (F10, F20, F30, F40, and F50 at 10%, 20%, 30%, 40%, and 50% elongation, respectively) were calculated. Moreover, the elongation at break (%ΔE) parameter was determined according to Equation (3) [27]:

%∆E = (Elongation at break − Initial length/Initial length) × 100.(3)

### 2.6. Assessment of the Rheological Interaction between ALG and PEOs 

In order to assess the occurrence of an interaction between ALG and PEOs, blends containing ALG 1% *w*/*w* in association with (i) l-PEO 2.2% *w*/*w*, (ii) h-PEO 1.5% *w*/*w*, or (iii) l-PEO 1.98% *w*/*w*/h-PEO 0.15% *w*/*w*, without surfactants, were subjected to viscoelastic measurements by means of a rotational rheometer (MCR 102, Anton Paar, Turin, Italy) equipped with a cone plate combination (CP50-1, ∅ = 50 mm; θ = 1°) as measuring system. Solutions of the individual polymers, having the same concentrations as in the blends, were subjected to the same characterization. All measurements were carried out at 33 °C after a rest time of 1 min.

Stress sweep and oscillation tests were performed as previously described. In particular, a shear stress, chosen in the linear viscoelastic region, was applied at increasing frequencies (1–20 Hz); the elastic response of the sample was measured as a function of frequency.

The interaction parameter ΔG′ was calculated at 1 Hz according to the following equation [28]:

ΔG′ = G′_mix_ − (G′_ALG_ + G′_PEO_) (4)

where

G′_mix_ = storage modulus (Pa) of ALG/PEO blends: 1% *w*/*w* ALG/2.2% *w*/*w* l-PEO; 1% *w*/*w* ALG/1.5% *w*/*w* h-PEO; 1% *w*/*w* ALG/1.98% *w*/*w* l-PEO/0.15% *w*/*w* h-PEO.

G′_ALG_ + G′_PEO_ = theoretical value (Pa) calculated as the sum of G′ values of individual components, having the same concentration as in the blends: ALG 1% *w*/*w*; 2.2% *w*/*w* l-PEO; 1.5% *w*/*w* h-PEO; 1.98% *w*/*w* l-PEO/0.15% *w*/*w* h-PEO.

Since the two grades of PEO are characterized by different viscoelastic properties, in order to compare their interaction with ALG on a homogenous basis, in the present work it was proposed to employ the normalized parameter ΔG′/G′, calculated by normalizing ΔG′ value for the theoretical value.

### 2.7. Statistical Analysis

Whenever possible, experimental values of the various types of measurements were subjected to statistical analysis carried out using the statistical package Statgraphics 5.0 (Statistical Graphics Corporation, Rockville, MD, USA). In particular, one-way ANOVA—Multiple Range Test was used.

## 3. Results and Discussion

### 3.1. Characterization of the Polymer Solutions for Electrospinning

Electrospinning is a simple and versatile technique that provides submicron fibers with different morphology from a polymeric solution, even just by modulating polymer concentration or MW, which, in turn, affects most of the rheological and electrical solution properties [12].

ALG, as many other polysaccharides, can be electrospun only after blending with other spinnable polymers due to its intrinsic properties, such as its low sol–gel transition concentration and its rigid and extended worm-like molecular structure rich in polyanion chains.

It is well known that, at a given polymer concentration, the chain entanglement density (number of entanglements per unit volume) increases with increasing MW; accordingly, the viscosity of the polymer solution increases [16]. It follows that if, at a given concentration, low-MW polymer solutions lead to the formation of non-uniform or beaded fibers, the use of high-MW polymers instead guarantees a sufficient number of chain entanglements to generate a continuous jet during electrospinning and, thus, to produce homogenous fibers with a larger mean diameter [29]. Furthermore, it should be remembered that, at a certain polymer MW, a minimum solution concentration, known as the critical entanglement concentration (CEC), is required to obtain bead-free fibers [25,30].

**Figure 1:**
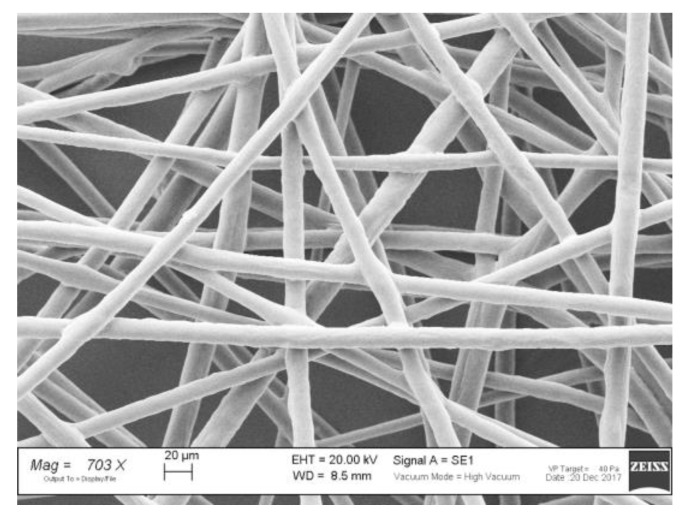
SEM micrograph of ALG/h-PEO fibers.

In a previous work of ours [26], ALG was blended with high-MW PEO (h-PEO, M_W_ = 4000 kDa) to obtain a polymer aqueous solution able to produce homogenous fibers by electrospinning (Figure 1). The optimized ALG/h-PEO blend was composed of 1% *w*/*w* ALG and 1.5% *w*/*w* h-PEO. The resulting h-PEO concentration associated with ALG was 15 times higher than the polymer CEC, which was 0.1% *w*/*w* (see Supplementary Data, Figure S1). The fibers obtained were characterized by a size of the order of microns.

In the present work, low-MW PEO (l-PEO, MW = 600 kDa) and its mixture with h-PEO were used to enhance ALG electrospinnability, instead of h-PEO.

**Figure 2:**
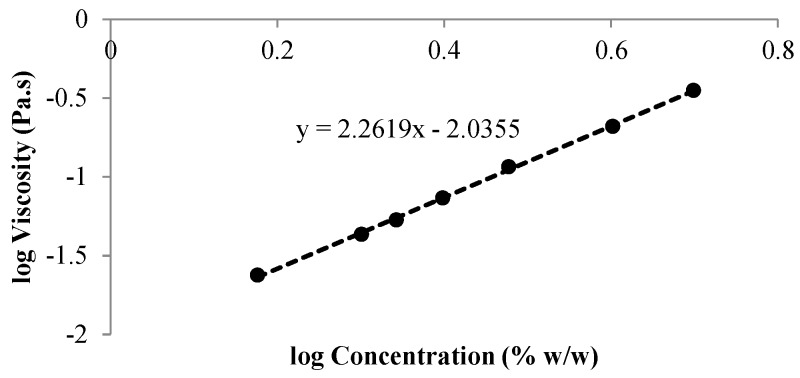
Log–log profiles of viscosity at 1000 s^−1^ versus l-PEO concentration (% *w*/*w*).

At first, a rheological study was performed to identify the concentration of l-PEO that showed, when dissolved in deionized water, a viscosity value at high shear rate (1000 s^−1^) equal to that of an aqueous solution of h-PEO at 1.5% *w*/*w*, which is the concentration identified as optimal to prepare an ALG/h-PEO solution able to produce homogenous fibers [26]. In particular, the viscosity values of l-PEO solutions prepared at increasing concentrations ranging from 1.5 to 5% *w*/*w* were measured and plotted versus l-PEO concentrations on a log–log scale (Figure 2). The experimental data were fitted with a linear regression and the l-PEO concentration having the same viscosity as 1.5% *w*/*w* h-PEO was identified by extrapolation. The resulting concentration was 2.2% *w*/*w*, which is higher than l-PEO CEC (equal to 1.3% *w*/*w*, see Supplementary Data, Figure S2).

**Figure 3:**
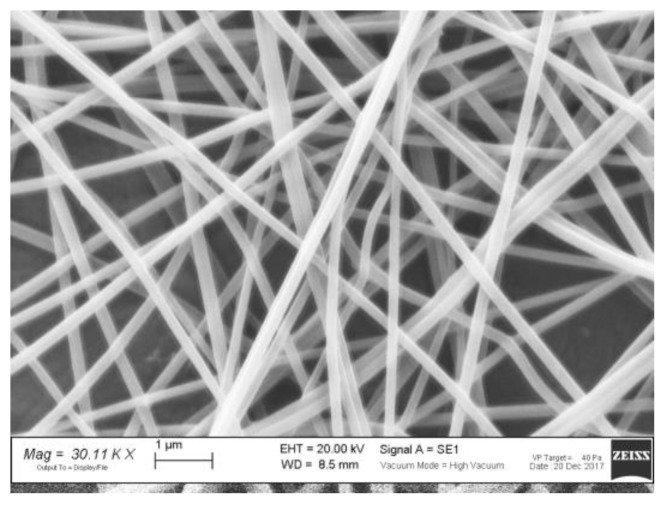
SEM micrograph of ALG/l-PEO fibers.

Therefore, l-PEO was blended with ALG in order to achieve concentrations equal to 2.2% and 1% *w*/*w*, respectively. Figure 3 shows that such an ALG/l-PEO solution, when electrospun, produces homogenous fibers. As expected, the use of l-PEO was responsible for the production of fibers having a smaller size (0.213 ± 0.005 μm, *n* = 30) than those obtained from the ALG/h-PEO blend (13.3 ± 1.9 μm, *n* = 30).

Such a result could be explained by considering the time required by PEO chains to extend in the distance between the spinneret and the collector. The l-PEO chains, being short, take less time to elongate and extend in water than do the h-PEO ones; the l-PEO aqueous jet has the necessary time to stretch until it reaches a nano-diameter before leaving dried fibers on the collector. The length of h-PEO chains, instead, slows down the elongation phenomenon, and when the jet reaches the collector, it still has micrometric dimensions [16,31].

Seven mixtures (Ms) of ALG/h-PEO/l-PEO (M1–M7) containing different concentrations of the two PEO grades were prepared in order to evaluate if the combined use of h-PEO and l-PEO could modulate/improve the morphological and mechanical properties of the ALG-containing fibers, with respect to those obtained from ALG/h-PEO and ALG/l-PEO blends. The concentrations of both h-PEO and l-PEO in each M were defined so that each h-PEO/l-PEO combination would show the same viscosity at high shear rate (1000 s^−1^) as 1.5% *w*/*w* h-PEO aqueous solutions (a viscosity equal to that of 2.2% *w*/*w* l-PEO).

Therefore, each mixture (M) was characterized in terms of viscoelasticity and conductivity.

H, L, and all Ms were subjected to dynamic viscoelastic measurements in order to study the contribution of each PEO to the mixture viscoelastic properties.

**Figure 4:**
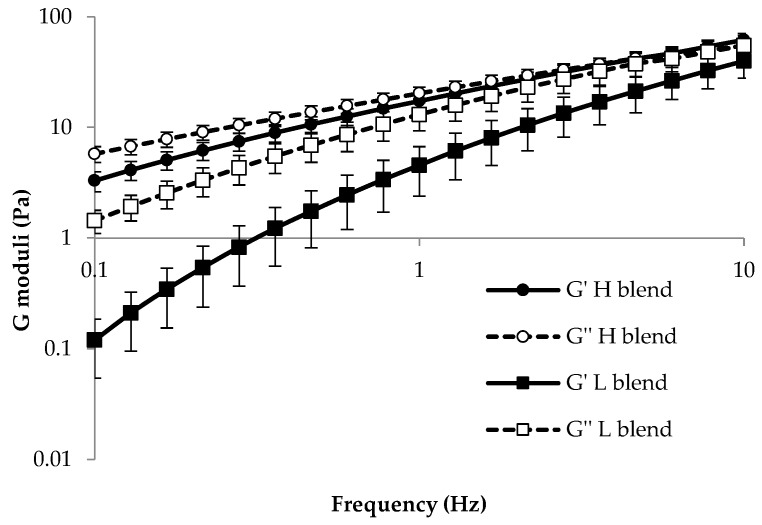
G′ and G″ profiles of H and L (mean values ± SD.; *n* = 3).

The profiles of storage (G′) and loss (G″) moduli obtained for H and L are reported in Figure 4. It can be observed that for both H and L, G′ and G″ moduli increase on increasing frequency; this trend is typical of polymeric solutions. Moreover, H is characterized by higher values of both moduli with respect to L. The two blends show different behaviour: L is characterized by greater viscous properties with respect to elastic ones in the whole range of frequencies considered, as demonstrated by a higher G″ profile with respect to the G′ one. On the contrary, a crossover point is evident in the G′ and G″ profiles of H. In particular, G″ values lower than G′ ones are observed for frequencies higher than 2.8 Hz. It is possible to clarify such behaviour by using a mechanical model based on a combination of springs (elastic elements) and dashpots (viscous elements). At high frequency values, the springs can elongate and contract, while the dashpots have very little time to move. For this reason, at low frequency values, the elastic-solid-like behavior prevails. On the contrary, at low frequency values, prevalence of the viscous-fluid-like behavior occurs; the springs extend but the dashpots have more time to move, and their extension greatly exceeds those of the springs. For L, the crossover point occurs at frequencies higher than 10; this is probably due to a reduced number of entanglements caused by the low polymer MW. Analogously to L, all Ms show no crossover point in the frequency range investigated (data not shown).

**Figure 5:**
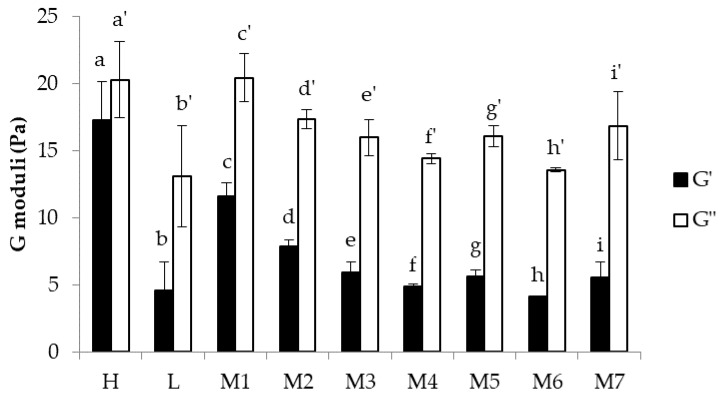
Values of G′ and G″ moduli measured at 1 Hz for H, L, and Ms (mean values ± SD; *n* = 3). One-way ANOVA; Multiple Range Test (*p* < 0.05): a vs. b–i; b vs. c,d; c vs. d–i; d vs. e–i; a’ vs. b’, e’–i’; b’ vs. c’, d’, i’; c’ vs. d’–h’; d’ vs. h’; h’ vs. i’.

In Figure 5, G′ and G″ values measured at 1 Hz of H, L, and Ms are compared. All Ms show a behaviour similar to that of L, being characterized by G″ values significantly higher than G′ ones. For H, instead, no significant differences are evidenced between G′ and G″ values. Moreover, a significant (*p* < 0.05) increase in G′ value is observed for h-PEO concentrations higher than 0.25% *w*/*w* (L vs. M1, M2). For such concentrations, the increase in the chain entanglement degree due to h-PEO is responsible for an increase in elasticity. It can be observed that M1 and M2 are characterized by G′ values significantly lower than the G′ value observed for H, while G″ does not change moving from H to M1 and M2. Such results point out that, among the elastic and viscous components, the elastic one is more sensitive to the presence of l-PEO.

**Figure 6:**
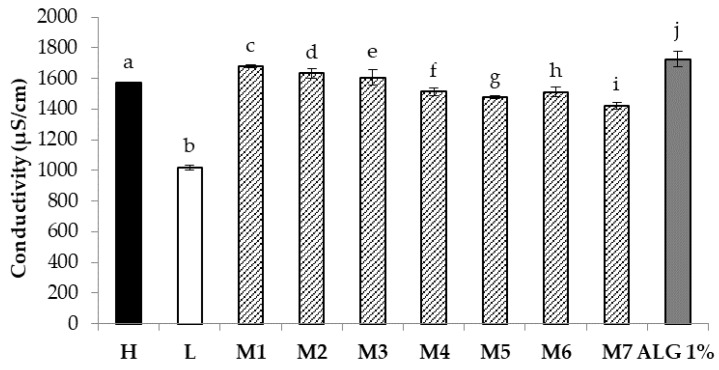
Conductivity values of H, L, and Ms (mean values ± SD; *n* = 3); one-way ANOVA; Multiple Range Test (*p* < 0.05): a vs. b–d, f–j; b vs. c–j; c vs. d–j; d vs. f–j; e vs. f–j; f vs. g–j; g vs. h–j; h vs. j; i vs. j.

The conductivity of H, L, and Ms was also assessed (Figure 6). All Ms show conductivity values significantly lower than an ALG aqueous solution prepared at the same concentration (1% *w*/*w*) present in the mixtures, suggesting that PEO has the ability to shield ALG negative charges. In particular, all Ms are characterized by conductivity values significantly (*p* < 0.05) higher than that of the L blend, suggesting that the presence of PEO at high molecular weight, even if at low concentrations, strongly influences the mixture electrical resistance. The results obtained point out that PEO shows a different capability to shield ALG negative charges depending on polymer molecular weight. In particular, it can be hypothesized that short chains, being characterized by high mobility in solution, are able to better wrap ALG negative chains, showing a greater shield effect.

As already mentioned, it is well known that in order to form uniform and bead-free fibers, polymer concentrations higher than the CEC have to be used. On this basis, it seemed interesting to evaluate for each polymer grade present in the mixtures the ratio between the polymer concentration and the CEC.

**Figure 7:**
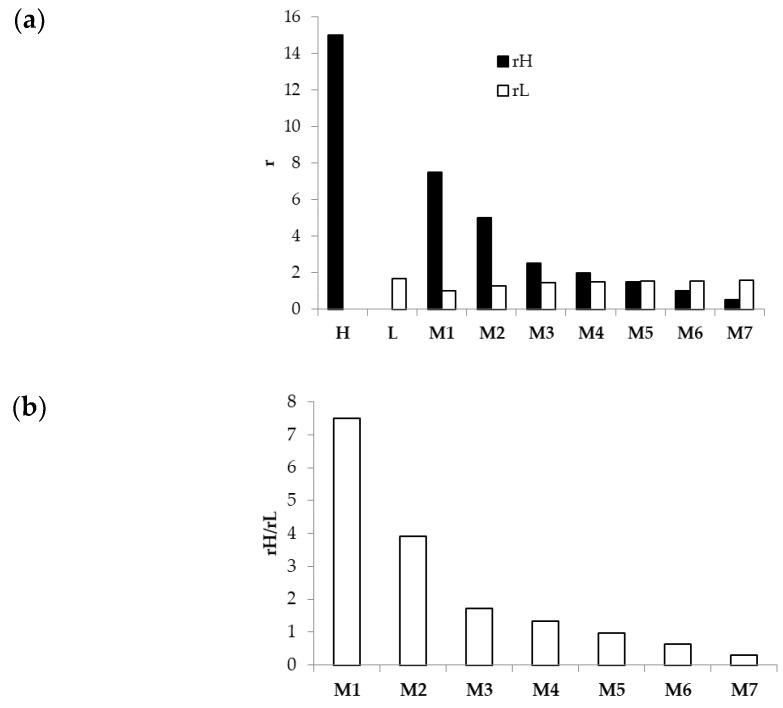
(**a**) rH and rL values calculated for H, L, and Ms; (**b**) rH/rL ratio calculated for all Ms.

Therefore, two parameters, rH and rL, were calculated for each M. These parameters indicate whether the PEO concentration employed in each M could guarantee the formation of chain entanglements, which are crucial to electrospinning. For h-PEO, only M7 shows a rH lower than 1, whereas all Ms are characterized by rL values of at least 1, indicating that, in all the mixtures, l-PEO was present at a concentration greater than or equal to the CEC (Figure 7a), a necessary condition for chain entanglements.

For each M, the ratio rH/rL was also calculated in order to better identify which of the two PEO grades prevails over the other in terms of exceeding the CEC and, thus, in its capability to form chain entanglements. Figure 7b demonstrates that M1, M2, M3, and M4 are characterized by a prevalence of h-PEO contribution over the l-PEO one; on the contrary, l-PEO prevails in the case of M6 and M7. A balance between l-PEO and h-PEO contributions occurs for M5, being characterized by a rH/rL value very close to 1.

### 3.2. Characterization of Electrospun Fibers

All Ms are able to produce fibers by electrospinning, regardless of process conditions. Since such mixtures have been prepared by maintaining constant the viscosity at high shear rate (1000 s^−1^), this result points out the importance of this parameter.

Table 2 reports the sizes of the fibers obtained from H, L, and Ms after electrospinning under four different process conditions, together with the rH/rL values. It is evident that fiber morphology results were differently affected by the experimental conditions, depending on mixture composition and rH/rL values.

At 15 cm and 15 kV, all Ms, with the exception of M7, produce fibers with a size in the order of microns.

At 20 cm and 20 kV, M5, M6, and M7, which are characterized by rH/rL values of <1, originate fibers with a mean diameter lower than 1 μm.

At 20 cm and 25 kV, M3, M4, M5, M6, and M7 produce fibers with a mean diameter lower than 1 μm. An increase in the applied voltage during the electrospinning process leads to a decrease in fiber dimension for M3 and M4 that, when electrospun at the same spinneret–collector distance but at a lower voltage (20 kV), originates micro-scale fibers.

At 25 cm and 25 kV, all Ms, with the exception of M1 and M2, which are characterized by rH/rL ≥ 4, originate fibers with a mean diameter lower than 1 μm.

To clarify how the process conditions could influence the morphology of the fibers obtained from the mixtures composed by different grades of the same polymer, it was decided to focus our attention on four mixtures characterized by different rH/rL values, namely, M1, M3, M5, and M7.

**Figure 8:**
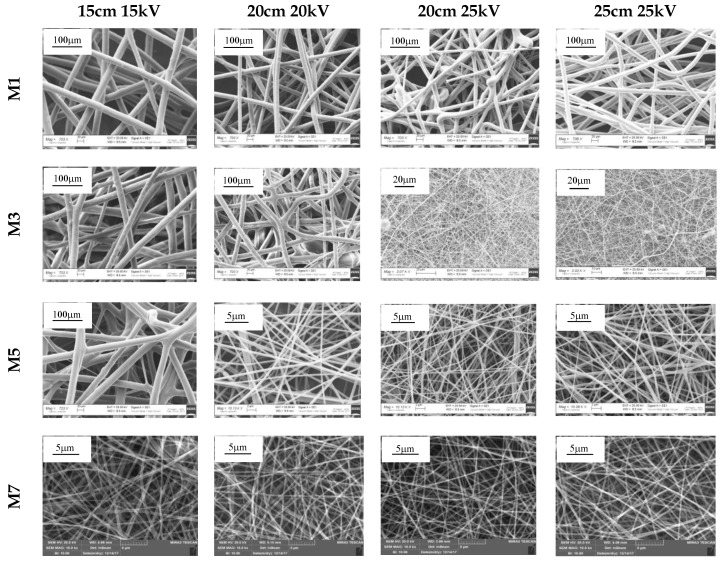
SEM micrographs of M1, M3, M5, and M7 fibers.

In Figure 8, SEM micrographs of the abovementioned fibers are reported.

**Figure 9:**
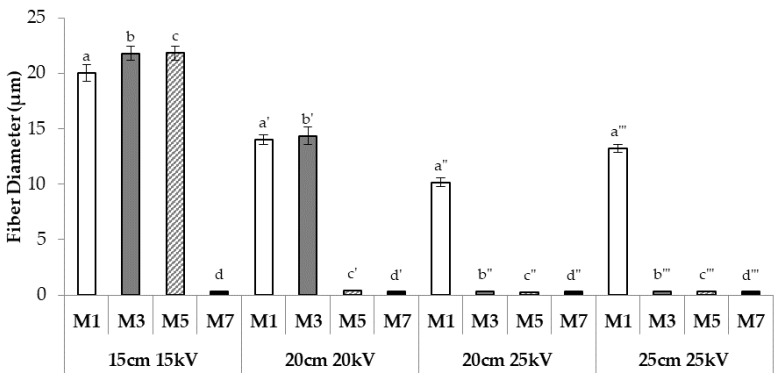
Dimensions of fibers obtained from M1, M3, M5, and M7 when electrospun under four different process conditions in terms of spinneret–collector distance and applied voltage (mean values ± SE; *n* = 30); one-way ANOVA; Multiple Range Test (*p* < 0.05): a vs. a’–a’’’, a’ vs. a’’, a’’ vs. a’’’; b vs. b’–b’’’, b’ vs. b’’, b’’’; c vs. c’–c’’’; d vs. d’–d’’’.

Figure 9 shows the diameters of the fibers obtained from M1, M3, M5, and M7 after electrospinning under four different process conditions.

Maintaining the same spinneret–collector distance, an increase in the applied voltage from 20 to 25 kV leads to a decrease in the size of the fibers obtained from M1. Regardless of the process conditions, M1 fibers are characterized by a mean diameter of ≥10 μm, suggesting the prevalence of h-PEO behaviour over that of l-PEO. This result is consistent with the high rH/rL value (7.5) that was calculated for M1.

As for M3, a decrease in fiber dimension is observed when the mixture is electrospun at the same spinneret–collector distance (20 cm) but at a higher voltage: in contrast to what was observed for M1, a reduction in fiber dimension from micro- to nano-scale is observed with increasing voltage. No significant differences in fiber diameter are evidenced with increasing distance from 20 to 25 cm. For this mixture, characterized by a rH/rL value (1.72) much lower than that of M1, fiber dimensions are strictly related to the process conditions set up: an increase in the applied voltage, from 20 to 25 kV, induces a variation in fiber dimension of two orders of magnitude.

For M5, characterized by a rH/rL parameter close to 1, both a voltage of >15 kV and/or a distance of >15 cm are responsible for the production of nano-fibers.

Finally, M7, characterized by the lowest rH/rL value, produces fibers with nano-scale dimension, independently of process conditions.

It is generally recognized that the distance between needle tip and collector and the applied voltage affect fiber morphology [12]. In particular, some authors proved that a critical spinneret–collector distance is needed to obtain homogeneous fibers [11]. In fact, the distance is a crucial parameter for modulating fiber deposition time and solvent evaporation rate [32]. Large-sized fibers formed for small distance values, whereas a decrease of fiber diameter was observed on increasing distance [32,33]. The applied voltage also affects fiber morphology: an increase in this parameter results in the formation of smaller-diameter fibers, due to a greater stretching of the polymer solution in correlation with the charge repulsion with the polymer jet [13].

The influence of both these experimental conditions (distance and applied voltage) varies according to intrinsic polymer properties such as molecular weight and, in the case of mixtures of two different polymer grades, the ratio between the two components.

The results so far obtained point out that the rH/rL values are predictive of the influence of the experimental condition on fiber size. In fact, for high rH/rL values (≥3.9; M1 and M2), fibers present micro-scale size independently of experimental conditions, as observed for H fibers containing only h-PEO. Analogously, for low rH/rL values (<0.5; M7), fibers characterized by nano-scale size are obtained at the various distance/voltage parameters, showing the same behaviour as L fibers based on l-PEO only. Intermediate rH/rL values are related to fiber dimensions markedly affected by electrospinning conditions: fibers are characterized by a micro-scale size at low distance/voltage values and, vice versa, by a nano-scale size at high distance/voltage values. 

It is well known that the mechanical properties of the electrospun fibers could be affected by several parameters, such as the solution composition, concentration, and possible interactions between the polymers [18]. Therefore, it seemed interesting to investigate how the mixture composition, strictly related to fiber dimensions, could affect the mechanical properties of the fibers. In particular, the contribution of both h-PEO and l-PEO to the tensile strength and elongation of the fibers was evaluated through a tensile test: M1, M3, M5, and M7 fibers obtained by electrospinning under the same process conditions (20 cm and 20 kV) were compared to H and L ones. The above mentioned spinneret–collector distance and applied voltage were chosen since they have proved to be the process conditions able to produce the most homogeneous fibers for all the mixtures considered.

**Figure 10:**
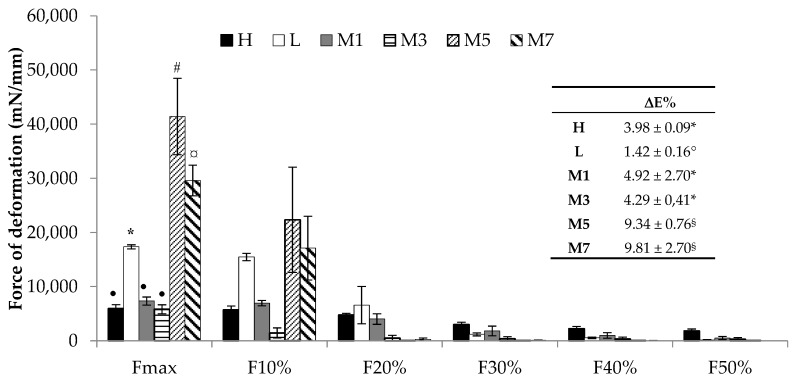
Mechanical properties of H, L, M1, M3, M5, and M7 fibers: maximum force of deformation and forces measured at different deformation values are reported (mean values ± SE; *n* = 3). Different symbols indicate statistically different data (*p* < 0.05; one-way ANOVA; Multiple Range Test). In the inset, elongation at break (ΔE%) values of electrospun fibers obtained from H, L, and M solutions are reported (mean value ± SE; *n* = 3).

The maximum force of deformation and forces at different deformation values were measured for M1, M3, M5, and M7 fibers in comparison with H and L ones. Figure 10 demonstrates that M5 fibers are characterized by the highest mechanical resistance, followed, in decreasing order, by M7, M1, and M3. This comparison reveals that the fibers with a nano-scale diameter (M5 and M7) show values of maximum force of deformation significantly higher than those measured for the fibers with a mean diameter of >10 μm (M1 and M3). The latter are also characterized by an elastic deformation, which is observed also for H and L fibers and is lacking in M5 and M7 fibers; Figure 10 shows an evident break of these membranes, as indicated by the zero force values for deformations higher than 10%. The results obtained are in line with those reported in the literature [34,35]. In particular, Tan and Lim (2006) observed that fiber mechanical properties depend on fiber diameter: smaller diameters result in higher strength and lower ductility [34]. Wang et al. (2015) demonstrated that a decrease in fiber diameter was responsible for an increase in fiber density that, in turn, produced an improved fiber mechanical strength [35].

Moreover, focusing on the maximum force of deformation values, it is evident that the substitution of an amount of h-PEO (up to 1.25% *w*/*w*) with l-PEO (H vs. M1, M3) does not induce any significant variations in the fiber mechanical resistance. This could be due to the fact that all these fibers are characterized by a micro-scale size. On the contrary, the substitution of an amount of l-PEO (up to 0.2% *w*/*w*) with h-PEO (L vs. M5, M7) is responsible for a significant increase in fiber mechanical strength. Since such fibers are characterized by similar nano-scale dimensions, this behaviour is attributable to different fiber composition: the increase in h-PEO concentration does not affect fiber size but is responsible for an increase in their mechanical resistance.

The fibers that show the highest mechanical resistance are M5, which are characterized by nano-size diameter and by a rH/rL value close to 1.

As for elongation at break, it can be observed that the nano-scale fibers (M5 and M7) are characterized by the highest capability to elongate when subjected to a tensile force. According to the literature [34], L fibers are characterized by a lower elongation at break (ΔE%) value with respect to H fibers, indicating a lower ductility. The highest ΔE% values, observed for M5 and M7, are attributable to the presence of h-PEO (Figure 10).

### 3.3. ALG/PEO Rheological Interaction

It is known that PEO, when mixed with ALG, leads to the formation of intermolecular hydrogen bonds. These are responsible for an increase in the entanglement degree of the polymer solution that is functional in the formation of fibers by electrospinning [25,29]. To verify if the use of PEO at a different molecular weight could affect such an interaction, blends of ALG with l-PEO, h-PEO, or l-PEO/h-PEO were subjected to viscoelastic measurements, and the results were compared to those obtained for the individual ALG and PEO solutions, having the same concentrations as in the blends [28]. 

**Figure 11:**
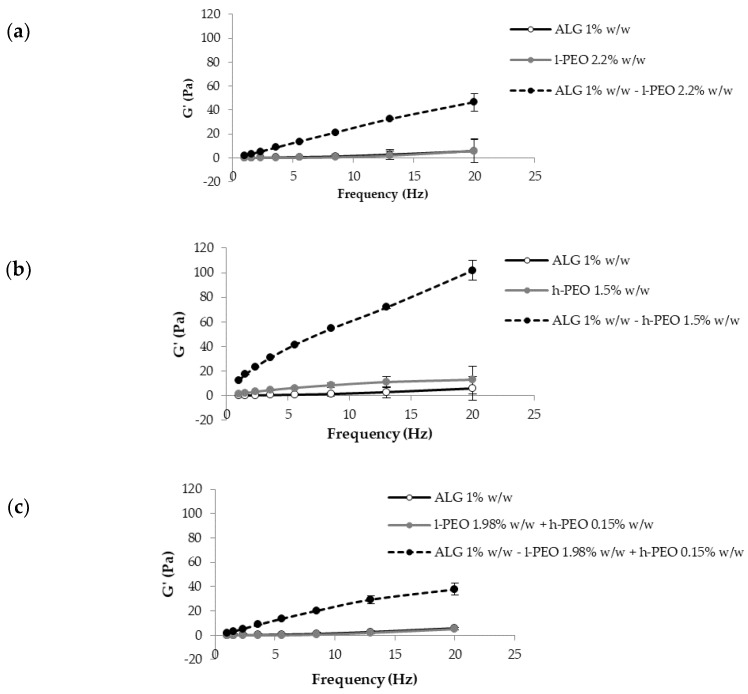
G′ vs. frequency profiles of blends containing ALG 1% *w*/*w* with (**a**) l-PEO 2.2% *w*/*w*, (**b**) h-PEO 1.5% *w*/*w*, and (**c**) l-PEO 1.98% *w*/*w*/h-PEO 0.15% *w*/*w* (mean values ± SD; *n* = 3).

In Figure 11, storage elastic modulus (G′) versus frequency profiles of aqueous ALG/l-PEO, ALG/h-PEO, and ALG/l-PEO/h-PEO blends in comparison with those obtained for ALG, l-PEO, h-PEO, and l-PEO/h-PEO aqueous solutions are reported. It can be observed that the blends are characterized by G′ values markedly higher than those of the individual polymer solutions.

**Figure 12:**
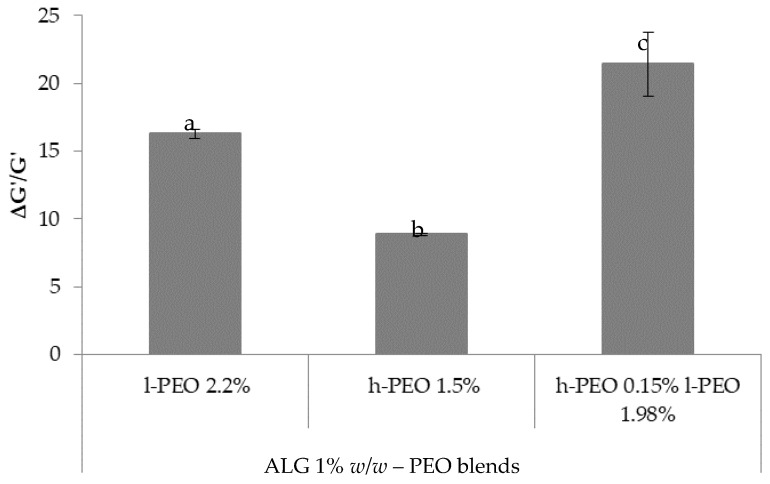
ΔG′/G′ values calculated for blends containing ALG 1% *w*/*w* with l-PEO 2.2%, h-PEO 1.5% and l-PEO 1.98%/h-PEO 0.15% (mean values ± SD; *n* = 3). One-way ANOVA, Multiple Range Test (*p* < 0.05); a vs. b, c; b vs. c.

Recently, some authors proposed to calculate the rheological interaction parameter ΔG′ as the difference between the G′ of the polymeric blend and the theoretical value, which is the sum of G′ values of the individual components, as an index of polymer/polymer interaction [28,36]. Since the two grades of PEO are characterized by different viscoelastic properties, in order to compare their interaction with ALG on a homogenous basis, in the present work it was proposed to employ the normalized parameter ΔG′/G′, calculated by normalizing ΔG′ values to the sum of G′ values of the individual solutions (Figure 12).

It can be observed that the presence in the blend of both PEO MW grades is responsible for higher ΔG′/G′ values, indicating an increase in the polymer capability to interact with ALG and then in the chain entanglement degree, crucial to the electrospinning process.

## 4. Conclusions

Among the different parameters considered for the characterization of the polymer mixtures, rH/rL, that indicates which of the two PEO (high or low MW) grades prevails over the other in terms of exceeding the CEC, has been proved to better explain the effect of the electrospinning conditions on fiber dimension. The addition of a small amount of h-PEO to l-PEO is responsible for a significant increase in fiber mechanical resistance, without affecting the nano-scale fiber size. Such behaviour is observed when rH/rL is in the range 0.3–1. Moreover, the mixing of the two PEO grades improves the interaction with ALG, resulting in an increase in chain entanglement degree that is crucial to the electrospinning process.

## Figures and Tables

**Table 1:** Composition of ALG (alginate)/h-PEO (high MW poly(ethylene oxide))/l-PEO (low MW poly(ethylene oxide)) mixtures, expressed as % *w*/*w*.

Mixtures	h-PEO	l-PEO	ALG	P407
M1	0.75	1.30	1	2
M2	0.50	1.66	1	2
M3	0.25	1.89	1	2
M4	0.20	1.94	1	2
M5	0.15	1.98	1	2
M6	0.10	2.02	1	2
M7	0.05	2.07	1	2

**Table 2:** Size (μm) of electrospun fibers obtained from H, L, and M solutions (mean value ± SE; *n* = 30).

	15 cm/15 kV	20 cm/20 kV	20 cm/25 kV	25 cm/25 kV	rH/rL
**H**	15.886 ± 0.810	13.732 ± 0.464	10.596 ± 0.482	12.304 ± 0.200	-
**L**	0.216 ± 0.060	0.213 ± 0.005	0.232 ± 0.012	0.206 ± 0.008	-
**M1**	20.047 ± 0.737	13.991 ± 0.431	10.177 ± 0.361	13.213 ± 0.328	7.50
**M2**	23.564 ± 0.994	15.378 ± 0.639	10.059 ± 0.390	12.164 ± 0.647	3.92
**M3**	21.794 ± 0.617	14.337 ± 0.786	0.359 ± 0.013	0.345 ± 0.018	1.72
**M4**	19.084 ± 0.874	15.275 ± 0.523	0.212 ± 0.016	0.423 ± 0.028	1.34
**M5**	21.818 ± 0.612	0.420 ± 0.011	0.253 ± 0.011	0.333 ± 0.013	0.98
**M6**	22.285 ± 1.068	0.294 ± 0.023	0.256 ± 0.015	0.283 ± 0.022	0.64
**M7**	0.344 ± 0.014	0.301 ± 0.01	0.298 ± 0.011	0.296 ± 0.009	0.31

## Supplementary Materials

The following are available online at http://www.mdpi.com/2079-4991/8/12/971/s1, Figure S1: Viscosity parameters (η_0_ and η_inf_) as a function of concentration (% *w*/*w*) of h-PEO. Log–log profiles are reported in the inset, Figure S2: Viscosity parameters (η_0_ and η_inf_) as a function of concentration (% *w*/*w*) of l-PEO. Log–log profiles are reported in the inset.
