# Supplementary material for: Electrospun Alginate Fibers: Mixing of Two Different Poly(ethylene oxide) Grades to Improve Fiber Functional Properties
Source: Nanomaterials (Basel). 2018 Nov 25;8(12):971. doi: 10.3390/nano8120971 (PMC6315736; doi:10.3390/nano8120971)
Supplement: Supplementary file 1 [file nanomaterials-08-00971-s001.pdf]

Supplementary Data

1. Assessment of critical entanglement concentration (CEC) of h-PEO and l-PEO

In the present study, h-PEO and l-PEO CECs were evaluated. Briefly, different h-PEO and l-PEO solutions with increasing polymer concentrations (ranging from 0.1 to 2% w/w and from 1.5% to 5% w/w respectively) were prepared in deionized water. Flow curves, reporting shear stress (τ) *versus* shear rate, were obtained for each PEO solution by means of a rotational rheometer (MCR102, Anton Paar, Turin, Italy), using a C50-1 cone (Ø = 50 mm and θ = 1°) as measuring system. Measurements were performed at increasing shear rates (10–1,000 s^{-1}) at 33°C (temperature to which the polymer solutions were subjected during the electrospinning process). Viscosity values at rest (η_0) and at high shear (η_{inf}) were calculated from the slopes of the straight lines that fitted the experimental data of the first and the second Newtonian zones of the flow curve. η_0 and η_{inf} were plotted versus the corresponding polymer concentration (% w/w) on bi-log scale. The intersection point of the two straight lines corresponded to CEC, Rossi et al. [1].

In Figure S1, the viscosity parameters η_0 and η_{inf}, indexes of h-PEO structure in deionized water at low and high shear rate, respectively, are reported against h-PEO concentration. Both the viscosity parameters exponentially increase on increasing h-PEO concentration. In the inset of Figure S1, log-log profiles of the viscosity parameters (η_0 and η_{inf}) *versus* l-PEO concentration allow the identification of the CEC, which corresponds to the intersection point between two straight lines that best represent, on a statistical basis, the experimental data. The CEC of h-PEO in deionized water results equal to 0.1% w/w.

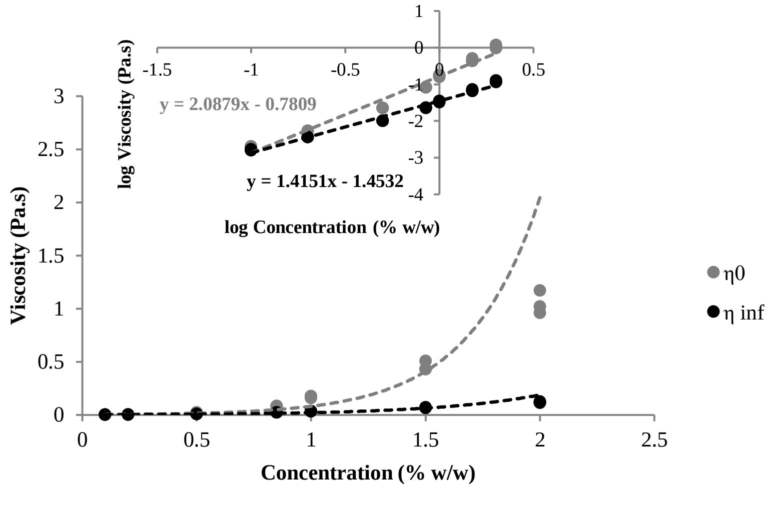

Figure S1. Viscosity parameters (η_0 and η_{inf}) as a function of concentration (% w/w) of h-PEO. Log–log profiles are reported in the inset.

In Figure S2, the viscosity parameters η_0 and η_{inf}, indexes of l-PEO structure in deionized water at low and high shear rate, respectively, are reported against l-PEO concentration. Both the viscosity parameters exponentially increase on increasing l-PEO concentration. In the inset of Figure S2, log-log profiles of the viscosity parameters (η_0 and η_{inf}) *versus* l-PEO concentration allow to identify the CEC of l-PEO, which results equal to 1.3% w/w.

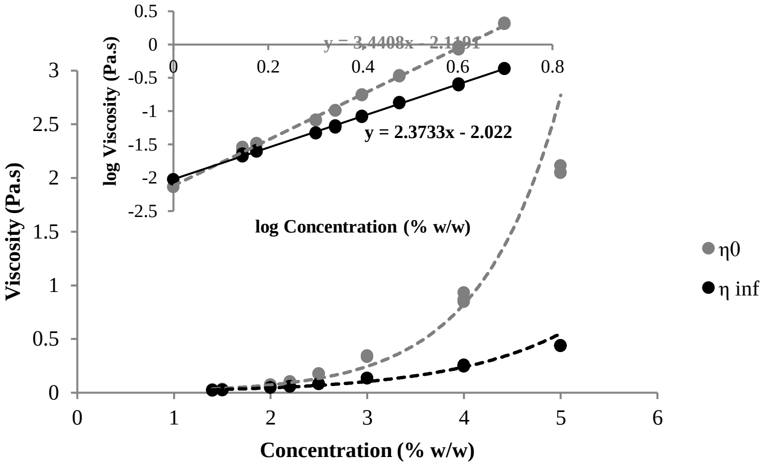

Figure S2. Viscosity parameters (η_0 and η_{inf}) as a function of concentration (% w/w) of I-PEO. Log–log profiles are reported in the inset.

References

1. Rossi, S.; Ferrari, F.; Bonferoni, M.C.; Caramella, C. Characterization of chitosan hydrochloride-mucin rheological interaction: influence of polymer concentration and polymer:mucin weight ratio. *Eur J. Pharm. Sci.* **2001**, *12*, 479–485.
